# Genomic Analyses Reveal the Common Occurrence and Complexity of *Plasmodium vivax* Relapses in Cambodia

**DOI:** 10.1128/mBio.01888-17

**Published:** 2018-01-23

**Authors:** Jean Popovici, Lindsey R. Friedrich, Saorin Kim, Sophalai Bin, Vorleak Run, Dysoley Lek, Matthew V. Cannon, Didier Menard, David Serre

**Affiliations:** a Malaria Molecular Epidemiology Unit, Institut Pasteur in Cambodia, Phnom Penh, Cambodia; b Genomic Medicine Institute, Cleveland Clinic, Cleveland, Ohio, USA; c National Center for Malaria Control, Phnom Penh, Cambodia; d Institute for Genome Sciences, University of Maryland School of Medicine, Baltimore, Maryland, USA; e Unité Biologie des Interactions Hôte-Parasite, Institut Pasteur, Paris, France; NIAID/NIH

**Keywords:** *Plasmodium vivax*, relapse, genomics, malaria

## Abstract

*Plasmodium vivax* parasites have a unique dormant stage that can cause relapses weeks or months after the initial infection. These dormant parasites are among the main challenges of vivax malaria control as they constitute a reservoir that is difficult to eliminate. Since field studies are confounded by reinfections and possible recrudescence of drug-resistant parasites, most analyses of *P. vivax* relapses have focused on travelers returning from regions of malaria endemicity. However, it is not clear whether these individuals accurately recapitulate the relapse patterns of repeatedly infected individuals residing in areas of endemicity. Here, we present analyses of vivax malaria patients enrolled in a tightly controlled field study in Cambodia. After antimalarial drug treatment was administered, we relocated 20 individuals to a nontransmission area and followed them for 60 days, with blood collection performed every second day. Our analyses reveal that 60% of the patients relapsed during the monitoring period. Using whole-genome sequencing and high-throughput genotyping, we showed that relapses in Cambodia are often polyclonal and that the relapsing parasites harbor various degrees of relatedness to the parasites present in the initial infection. Our analyses also showed that clone populations differed dynamically, with new clones emerging during the course of the relapsing infections. Overall, our study data show that it is possible to investigate the patterns, dynamics, and diversity of *P. vivax* relapses of individuals living in a region of malaria endemicity and reveal that *P. vivax* relapses are much more pervasive and complex than previously considered. (This study has been registered at ClinicalTrials.gov under registration no. NCT02118090.)

## IMPORTANCE

*P. vivax* parasites can remain dormant in the liver and relapse weeks or months after the initial infection, greatly complicating malaria control and elimination efforts. The few investigations of this dormant stage have relied on travelers and military personnel returning from areas of malaria endemicity. However, it is not clear whether these individuals, exposed to a limited number of infections, accurately represent the patterns of relapses of individuals living in areas of endemicity, who are repeatedly infected by *P. vivax* parasites. Our study combined tightly controlled fieldwork with comprehensive genomic analyses, and our report provides a first opportunity to investigate the patterns, dynamics, and diversity of *P. vivax* relapses directly with individuals living in areas of endemicity.

## INTRODUCTION

While ongoing efforts to eliminate malaria worldwide show promising results for *Plasmodium falciparum* malaria, the results for *P. vivax*, the main cause of human malaria outside Africa, are much less encouraging (1–4). One of the main challenges specific to vivax malaria is the existence of dormant parasites, i.e., hypnozoites. Hypnozoites can be released from the liver into the bloodstream weeks or months after the initial infection and cause relapses. This dormant stage is difficult to target as the hypnozoites can be eliminated only by treatment using primaquine, which can have dramatic side effects in G6PD (glucose-6-phosphate dehydrogenase)-deficient patients and is therefore difficult to deploy in many areas of endemicity (5, 6). In addition, hypnozoites further complicate malaria control by facilitating the geographic dispersion of *P. vivax* parasites and, at least theoretically, the acquisition of drug resistance by exposing parasites to a subtherapeutic level of antimalarial drugs.

Despite reports of fascinating recent studies performed using humanized mice (7) and sophisticated *in vitro* assays (8) that complemented seminal studies in human volunteers (9), we still know very little about this critically important *P. vivax* stage. In particular, studies of *P. vivax* relapses in patients are difficult to interpret as they are confounded by reinfections and possible recrudescence of drug-resistant parasites. As a consequence, studies of *P. vivax* relapses have often relied on data from infected travelers or soldiers returning from areas of endemicity (10–13). One potential concern with such studies is that these individuals likely had limited exposure to *P. vivax* and may therefore not accurately recapitulate the relapse patterns of individuals living in areas of endemicity. Here, we used a combination of genomic approaches to comprehensively characterize parasites from vivax malaria patients enrolled in a tightly controlled field study in Cambodia and to rigorously investigate the patterns, dynamics, and diversity of *P. vivax* relapses.

## RESULTS

### Relapses occur frequently in relocated patients cleared of *P. vivax* parasites after CQ treatment.

We recruited 20 *P. vivax*-infected patients in Cambodia and treated them with a standard, supervised course of chloroquine (CQ) (Nivaquine; Sanofi-Aventis, Paris, France). We then relocated these patients to an area with no transmission of malaria and monitored them for at least 60 days (see Materials and Methods for details).

**FIG 1:**
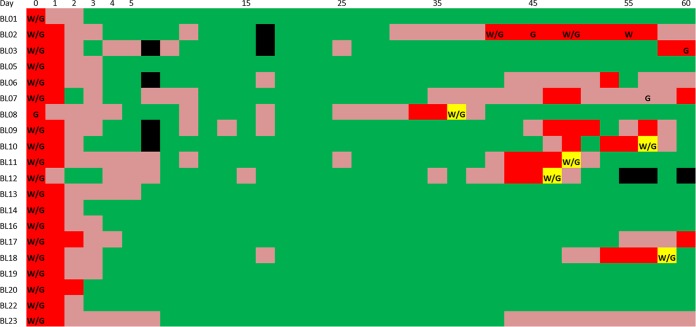
*P. vivax* positivity following chloroquine treatment. The figure shows the results of *P. vivax* detection following treatment (at day 0) for the patients (displayed as rows) for the 60-day monitoring period (*x* axis). Dark red indicates *P. vivax*-positive samples detected by both microscopy and qPCR, while light red indicates samples positive only by qPCR and green denotes *P. vivax-*negative samples. The yellow boxes indicate cases where a patient developed malaria symptoms and was retreated with chloroquine. "W" and "G" indicate instances where a blood samples was successfully characterized by *P. vivax* whole-genome sequencing (coverage of >50×) and genotyping (>50 SNPs), respectively. Black boxes indicate missing data.

All of the patients were clear of *P. vivax* parasites at 3 and 6 days after CQ treatment as measured, respectively, by microscopy and PCR (J. Popovici, L.R. Friedrich, S. Kim, A. Vantaux, S. Bin, V. Run, D. Lek, K.H. Hee, L.L. Soon-U, D. Serre, and D. Ménard, submitted for publication). However, among the 20 patients, 12 (60%) displayed recurrences of *P. vivax* parasites within 60 days (Fig. 1). In contrast, none of these relocated patients were positive for *P. falciparum* DNA, supporting the data indicating the absence of reinfections. Our data indicated that CQ efficiently eliminated all initial blood-stage parasites; while we detected isolated cases of *P. vivax* DNA-positive samples 1 week after treatment, we did not observe any microscopy-positive sample until day 33 (Fig. 1), when CQ levels were below therapeutic levels (<100 ng/ml [14]) (Popovici et al., submitted for publication). While it is impossible to irrefutably exclude the possibility of recrudescence of drug-resistant parasites (that may have persisted below detection level), our study relied on a very sensitive detection method and used tight monitoring (every second day), which reduced the possibility of false detections. In addition, CQ treatment of the five cases of symptomatic vivax malaria that occurred during the monitoring period led to the rapid elimination of all *P. vivax* parasites (Fig. 1), indicating that those parasites were not resistant.

Overall, our data indicated that the recurrence of the parasites detected weeks after CQ treatment was most likely caused by relapses of *P. vivax* hypnozoites from the liver rather than by reinfections or recrudescence of drug-resistant parasites.

### Recurring parasites often have genotypes different from the genotypes of the parasites present before treatment.

For five patients, we were able to generate high-coverage (>50×) genome sequencing data from the *P. vivax* parasites present in the initial infection and in the recurrence (see Table S1 in the supplemental material). These included parasites from four symptomatic recurring infections (corresponding to patients BL10, BL11, BL12, and BL18) and one asymptomatic recurring infection (patient BL02). To assess whether the recurring parasites were genetically identical to the parasites present before CQ treatment, we determined, for each patient, the numbers of *P. vivax* alleles that were detected in the recurrence but that had not been observed in the initial infection (referred to as “new alleles”; see also Materials and Methods). In all five cases, the recurring parasites displayed thousands of such new alleles (Table 1), indicating that at least some of the recurring parasites were not present initially.

10.1128/mBio.01888-17.5 TABLE S1  The table indicates the results of the whole-genome sequencing for each patient isolate, collected at enrollment (D0) or during relapse (DX). The number of total read pairs generated and number of read pairs mapped to the *P. vivax* reference genome sequence (and the percentage mapped) are indicated, as well as the mean genome coverage (Mean Cov.) and number of nucleotides sequenced at more than 50× coverage (Cov>50×). Download TABLE S1, PDF file, 0.2 MB. Copyright © 2018 Popovici et al. 2018 Popovici et al. This content is distributed under the terms of the Creative Commons Attribution 4.0 International license.

**TABLE 1:** Summary of the whole-genome sequencing and genotyping data^a^

Sample	Day of recurrence	Whole-genome sequencing					Genotyping			
		Cov D0	Cov DR	New alleles	Major all. diff	No. of clones (D0/DR)	Cov D0	Cov DR	New alleles	No. of clones (D0/DR)
BL02	D41	299	122	5,437	22,070	3/2	2,184	1,575	0/73	3/2
BL03	D60	313	—	—	—	1/—	2,921	1,425	0/87	1/1
BL07	D59/D57	271	***7***	—	—	3/—	2,472	403	5/51	3/3
BL08	D36R	***46***	171	—	—	—/2	2,713	1,572	1/96	3/1
BL10	D56RD0	205	177	35,400	38,185	1/1	1,546	1,956	22/86	1/1
BL11	D48RD0	153	377	4,442	30,002	3/1	3,438	1,797	1/96	3/2
BL12	D47RD0	431	146	19,930	33,466	3/3	2,292	1,334	18/101	3/3
BL18	D58R	374	163	3,614	5,580	1/1	2,155	1,145	6/82	1/2

^a^ The table indicates the average read coverage obtained for the samples collected at day 0 (“Cov D0”) and at recurrence (“Cov DR”) for the whole-genome sequencing data and genotyping data. The numbers highlighted in bold and italics indicate samples for which the coverage was considered too low for further analyses. The table also shows the number of alleles detected in the recurrent infection that were not observed in the initial infection (“New Alleles”) as well as the number of nucleotide positions where the major alleles were different in the initial and recurrent infections (“Major all. diff”). See Materials and Methods for details. —, not determined.

To confirm the genome sequencing results and to test whether the recurring clones that had not been detected in the initial infection might have been present at low abundance, we generated genotyping data with very high coverage (on average, greater than 1,500 × at day 0; Table 1). Since this genotyping assay relied on PCR amplification, we were able to characterize samples with lower levels of parasitemia than would have been possible by whole-genome sequencing and thus to analyze two additional asymptomatic recurrences (corresponding to patients BL03 and BL07). In addition, we were able to analyze one additional symptomatic recurrence for which we had failed to generate reliable whole-genome sequencing data from the initial infection (BL08). Overall, eight pairs of samples were successfully genotyped at more than 50 single nucleotide polymorphisms (SNPs). The genotypes and allele frequencies determined by high-throughput genotyping were highly concordant with those obtained by whole-genome sequencing (see, e.g., Fig. S1 in the supplemental material). In six of eight cases, we observed, in the recurring infections, alleles that were not detected in the primary infections (Table 1), confirming the genome sequence data and indicating that the recurrent infections typically included novel parasites. Note that the much lower number of new alleles observed in the genotyping data is due to the small number of nucleotide positions analyzed (at most, 101 SNPs) compared to more than 20 million nucleotides in the whole-genome sequencing data. These observations further supported the hypothesis that the recurrences of parasites were caused by relapses and not by recrudescence of chloroquine-resistant parasites.

10.1128/mBio.01888-17.1 FIG S1  Comparison of the allele frequencies determined by genotyping and whole-genome sequencing. Each dot represents one SNP and is displayed according to the allele frequency determined by genotyping (*x* axis) and whole-genome sequencing (*y* axis). The graph shows the data for the parasites present in BL02 at day 0. Download FIG S1, TIF file, 0.04 MB. Copyright © 2018 Popovici et al. 2018 Popovici et al. This content is distributed under the terms of the Creative Commons Attribution 4.0 International license.

**FIG 2:**
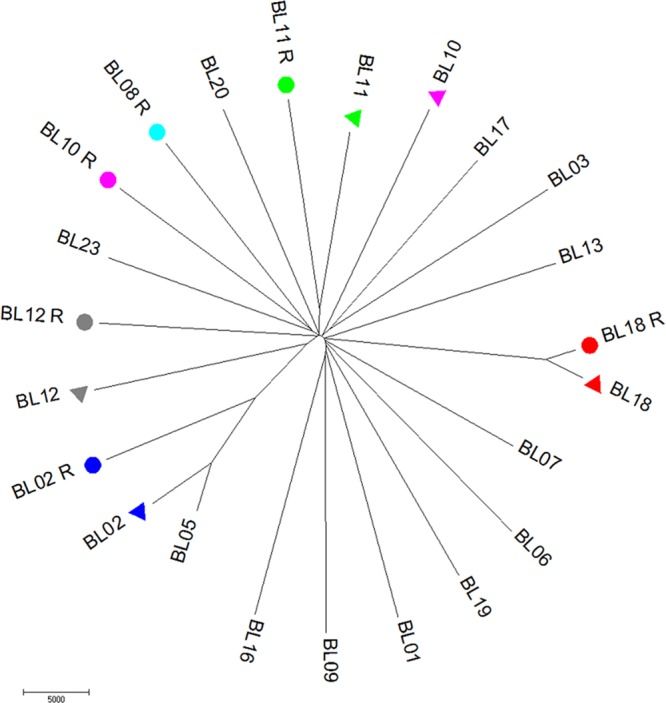
Genetic relationships among the dominant clones of each infection. The figure shows a neighbor-joining tree reconstructed using the number of nucleotide differences between the genomes of each clone. The parasites dominant in the recurring infections are highlighted with colored circles and the clones dominant in the corresponding primary infection with triangles of the same color. (The initial infection of BL08 is not included as the sequencing data did not fulfill our quality control [QC] criteria).

Polygenic infections complicate rigorous analyses of the genetic relationships among clones (15). To circumvent this issue, we initially limited our analyses to comparisons of the genomes of the most abundant clones seen with each infection. For all five cases for which we had genome data for the initial and recurrent infections, the dominant parasite in the initial infection was genetically different from the dominant parasite in the relapse by several thousand DNA polymorphisms (Table 1) and did not seem to be more closely related to it than to any other Cambodian *P. vivax* parasite (Fig. 2). One notable outlier was the dominant recurring parasite from patient BL18, which was different at only 5,580 SNPs from the initially dominant parasite (compared to 20,000 to 30,000 differences between any two unrelated parasites), suggesting that these clones might be closely related (also see below).

### *P. vivax* relapses are often polyclonal.

Previous studies have shown that *P. vivax* infections are often complex and contain multiple, genetically distinct clones (15–18). Consistent with these reports, we detected evidence of multiple clones in 10 of the 20 initial infections (i.e., before chloroquine treatment). More surprisingly, at least four of the eight relapses analyzed by whole-genome sequencing and genotyping also showed clear evidence of polyclonality (Table 1; a fifth relapse, that seen with patient BL18, showed a second, rare clone by genotyping but not by whole-genome sequencing). Indeed, some of these relapsing infections (e.g., that seen with patient BL12) displayed patterns of allele frequencies consistent with the presence of three or more clones (see, e.g., Fig. S2). Interestingly, the patients with polyclonal infections at the time of treatment were more likely to have polyclonal relapses than the patients with monoclonal infections, though this association did not reach significance (*P* = 0.07) and will need to be confirmed with a larger sample size.

10.1128/mBio.01888-17.2 FIG S2  Example of a polyclonal relapse. The figure shows the reference allele frequency plot for the parasites present in the relapse of BL12. Note the two peaks at ~50% and 5%, indicating that at least three parasites (representing approximately 47%, 47%, and 6% of the parasites sequenced) were present at the time of relapse. Download FIG S2, TIF file, 0.1 MB. Copyright © 2018 Popovici et al. 2018 Popovici et al. This content is distributed under the terms of the Creative Commons Attribution 4.0 International license.

### Relapsing parasites are often related to parasites present in the initial infection.

**FIG 3:**
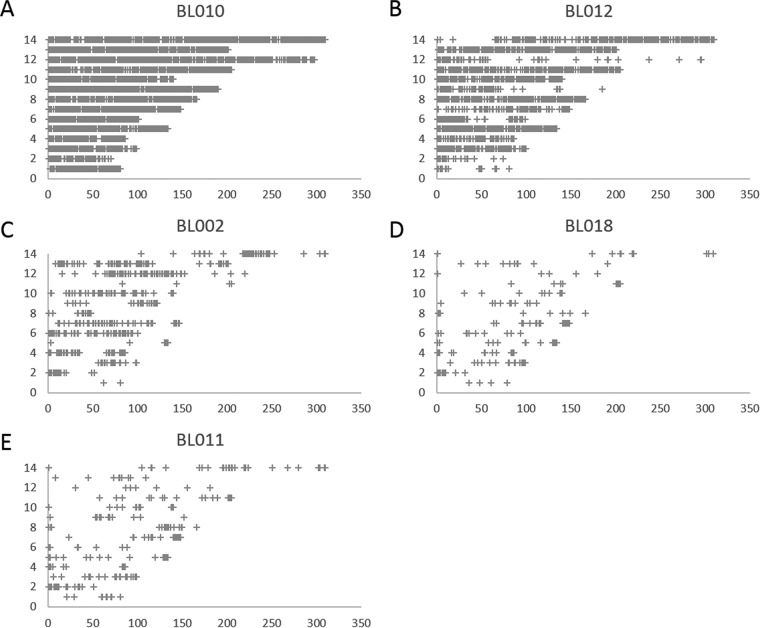
Relatedness between relapsing parasites and those present in the initial infection. The figure shows the distribution, throughout the genome, of the new alleles (corresponding to patients BL010 [A], BL012 [B], BL002 [C], BL018 [D], and BL011 [E]) that were detected in the relapse infection but not observed in the initial infection. Each cross represents a 10-kb window containing more than five new alleles and is displayed according to its genomic position (*x* axis, nucleotide position in kilobases; *y* axis, chromosome). For patient BL10, the new alleles are distributed throughout the genome, suggesting that the initial and relapsing parasites are unrelated (A), while for the other patients, some large chromosomic regions (e.g., most of chromosome 12 in BL12 [B]) are free of new alleles, indicating identical genetic makeup characteristics and close relatedness among the initial and relapsing clones.

Polyclonality prevents determining the genome sequence of each clone within an infection and, therefore, complicates the rigorous assessment of relatedness among clones. In this regard, it is noteworthy that the close relationship between the clones of BL18 (Fig. 2) was detected in monoclonal infections. To further investigate the relatedness between the parasites present in the initial infection and those present in the relapse of each patient, we analyzed the distribution, throughout the *P. vivax* genome, of new alleles observed in relapsing parasites but not detected in the initial infection. In one patient (BL10) who displayed both monoclonal initial and relapsing infections, these new alleles were distributed evenly throughout the genome, indicating that these two clones were unrelated (Fig. 3A). In all other patients, the new alleles clustered in genomic “blocks” (Fig. 3) consistent with meiotic siblings (10, 19); for example, the relapsing clones observed from patient BL12 must have been related to some of the clones initially present in the acute infection, as they carried very few new alleles throughout ~250 kb of chromosome 12 (though they differed significantly over the first 50 kb of this chromosome and throughout most of the genome [Fig. 3B]).

### Genetic monitoring of the relapsing parasites reveals complex temporal changes that occurred in clone populations during the course of the infection.

Since we collected blood samples from each patient every second day after treatment for at least 60 days, our study provided a unique opportunity to study dynamic changes in parasite composition and abundance during the course of the relapses (Table S2). While the low level of parasitemia seen at many time points limited the number of genotypes recovered, we were able to characterize genetic variations, corresponding to at least 20 SNPs, at three consecutive time points for five of the relapses (four symptomatic infections and one asymptomatic infection; Table S2). For these samples, we used the genotype information to identify differences in the numbers of clones detected (16) as well as to estimate variations in their relative proportions (see Fig. S3 for a theoretical example). In the relapse of patient BL18, we observed a single *P. vivax* clone at days 55, 56, and 57 but detected the presence of a second clone of low abundance at day 58, when the patient developed symptoms of malaria (Fig. S4). In the four other patients, we detected the presence of multiple clones in the earliest genotyped samples but the fates of these clones differed considerably. In patient BL11, the relative proportions of each clone seemed to remain constant throughout the infection. In patient BL10, the minor clone that was present at ~20% at days 53 and 55 was no longer detectable at day 56, when the patient became feverish (Fig. S4). Patients BL12 and BL02 displayed complicated patterns of allele frequencies that were consistent with variations in the relative abundances of the clones and the emergence of new genotypes (Fig. S4).

10.1128/mBio.01888-17.3 FIG S3  Schematic representation of the effect of temporal variation in clone populations on the allele frequencies. The figure shows examples of the type of changes in allele frequencies induced by a change in the clone proportion (middle row) or by the introduction of a new clone (bottom row), starting from an infection with either two clones (left) and three clones (right). Note that a change in the proportion of the clone populations typically leads to asymmetrical changes in allele frequencies (red arrow), while the introduction of new parasites is more clearly shown by the appearance of new alleles (green circles). Download FIG S3, TIF file, 0.2 MB. Copyright © 2018 Popovici et al. 2018 Popovici et al. This content is distributed under the terms of the Creative Commons Attribution 4.0 International license.

10.1128/mBio.01888-17.4 FIG S4  Changes in clone populations during the course of the relapses. The figure shows the allele frequencies determined for each genotyped SNP (blue dots) at two consecutive time points (shown on the *x* axis and the *y* axis, respectively). For example, the graphs show that a single clone was present in BL18 at day 56 and day 57 (left panel) but that a minor clone appeared at day 58 (as shown by the spread of allele frequencies of between 0% and 20% and between 80% to 100% on the *y* axis in the right panel). Similarly, two clones were present in BL10 and in similar proportions at day 53 and day 55 (~80% and 20%; left panel), but the minor clone was lost by day 56 (right panel). See also Fig. S3 for details. Download FIG S4, TIF file, 0.4 MB. Copyright © 2018 Popovici et al. 2018 Popovici et al. This content is distributed under the terms of the Creative Commons Attribution 4.0 International license.

10.1128/mBio.01888-17.6 TABLE S2  The table summarizes the results of the monitoring of relapsing infections. For each patient and each day following detection of *P. vivax* parasites, the table indicates the level of parasitemia estimated by qPCR and microscopy (expressed as the number of parasites per microliter), the number of SNPs successfully genotyped (i.e., coverage of >100×), and the number of clones estimated to be present based on the genotyping data (see Materials and Methods for details). Download TABLE S2, PDF file, 0.2 MB. Copyright © 2018 Popovici et al. 2018 Popovici et al. This content is distributed under the terms of the Creative Commons Attribution 4.0 International license.

**FIG 4:**
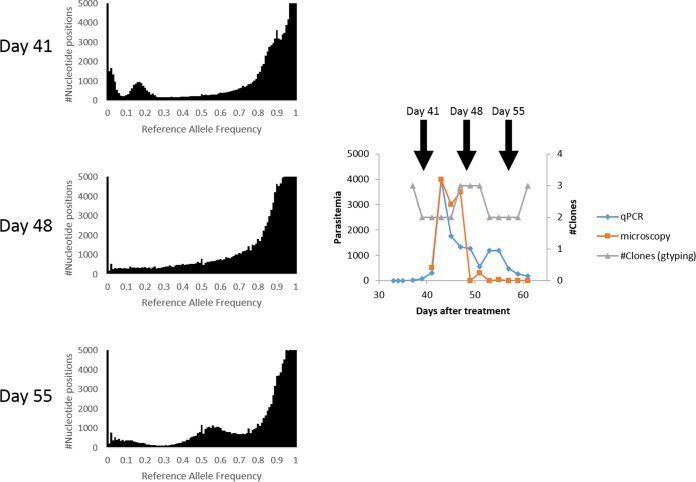
Complex changes in parasite populations detected by whole-genome sequencing. The figure shows the reference allele frequency plots generated from the parasites present in BL02 at days 41, 48, and 55 (left panel). Note the multiple modes at day 41 and day 55, indicating the presence of three main clones, while the lack of distinguishable modes at day 48 suggests the presence of either a single clone or numerous genetically different parasites in various proportions. These variations in the numbers of clones that were detected during the course of the infection are also reflected in the genotyping (gtyping) data generated from the same individual (gray curve, right panel) and accompany dramatic changes in parasitemia as determined by microscopy (orange curve) and qPCR (blue curve).

For one of the patients, BL02, who remained asymptomatic for 30 days during the monitoring period, we were able to complement the genotype data with whole-genome sequencing of the parasites at days 41, 48, and 55 (Table S1). These genome-wide data allowed us to more rigorously characterize changes in clone numbers and their proportions during the course of the infection (Fig. 4). The reference allele frequency (RAF) plot generated from parasites at day 41 was consistent with the presence of at least three clones in the infection: the dominant clone representing ~85% of the parasites, a second clone present at ~15%, and a third, minor clone (<5%). By day 48, the multiple modes in the distribution of RAF were not distinguishable, consistent with either (i) the presence of one single clone or (ii) a complex mixture of many clones with various levels of abundance. Interestingly, we observed at day 48 at least 8,500 alleles that were not detected at day 41, suggesting that several new clones appeared in the relapsing infection within that period, consistent with the results of the genotyping data (Fig. 4). By day 55, the distribution of RAF clearly showed multiple peaks again, consistent with the presence of fewer clones (two roughly equally abundant and at least one rare one), again mirroring the genotype data that showed a loss of a clone(s).

## DISCUSSION

Successful elimination of malaria worldwide is notably challenged by the difficulties encountered in our attempts to efficiently control *Plasmodium vivax*. While many factors contribute to the parasite’s resilience, the existence of a dormant stage in *P. vivax* plays a critical role, since it provides a reservoir of parasites that cannot easily be targeted using current approaches (20). Unfortunately, the biology of this dormant stage remains largely mysterious as patient studies are confounded by reinfections and the difficulties encountered in attempts to differentiate true relapses from possible parasite recrudescence after treatment. Several studies have circumvented these issues by analyzing travelers and military personnel returning from areas of endemicity (10–13, 21). However, it is unclear whether the relapses analyzed in these studies adequately represent those occurring in individuals living in areas of malaria endemicity, who are likely to have been infected repeatedly with *P. vivax* parasites and might therefore carry a much larger number of hypnozoites. Here, we describe the results of genomic analyses of a tightly controlled field study that enabled investigating relapses from individuals residing in a region of malaria endemicity. First, we relocated patients to an area with no malaria transmission for the entire duration of the study (60 days) to exclude possible reinfections. Second, we ruled out the possibility that recurring infections were caused by recrudescence of drug-resistant parasites by (i) testing for *P. vivax* DNA in patient blood every second day after treatment using a highly sensitive detection method, (ii) comparing the genotypes of the parasites in the initial infections and recurrences, (iii) verifying that drugs in patients’ blood on the day of recurrence were below therapeutic levels, and (iv) successfully treating symptomatic recurrences with another full course of chloroquine (Popovici et al., submitted for publication).

Using this rigorously controlled setting, we observed that *P. vivax* relapses were pervasive among patients living in a low-transmission area of Cambodia; among the 20 patients enrolled in this study, 12 (60%) relapsed within 60 days (or, more exactly, within the last 30 days of our study, once CQ concentrations dropped below the therapeutic level). While only five of these patients developed symptoms of malaria from these recurring infections, our results emphasize the importance of relapses for malaria control; without radical cure, these patients may present opportunities for further transmission of the disease despite the effective treatment against blood stage parasites (22). Indeed, we observed, by microscopy, gametocytes for 7 of these 12 relapses. Our genomic analyses also confirmed previous observations (10, 11, 13) that relapsing parasites are sometimes meiotic siblings of those causing the preceding acute infection; for several infections, the patterns of genetic diversity clearly indicated that the parasite(s) causing the relapse shared extended haplotypes with some of the parasites present initially and was likely to have been transmitted by the same mosquito (where the meiotic recombination occurred). While this high relatedness is not surprising in relapses of travelers who have likely been exposed to a small number of infected bites, it is interesting to observe the same phenomenon in individuals living in areas of endemicity. This finding also suggests that, for a significant proportion of infections, one clinical infection will likely be followed by the reactivation of dormant parasites. On the other hand, and perhaps more surprisingly, we observed that many relapses were polyclonal and often contained three or more different *P. vivax* clones. We also noted that patients presenting polyclonal infections before treatment tended to display polyclonal relapses (though this association did not reach statistical significance). One hypothesis for explaining this observation is that patients with polyclonal infections had higher levels of previous exposure to *P. vivax* and, therefore, carried a greater load of hypnozoites in their liver (and, possibly, that releases of hypnozoites from these earlier infections contributed to their polyclonality at day 0). This hypothesis is also consistent with the observation that some of these relapsing clones were not detected at day 0 and were not related to those initially present, indicating that they likely originated from infections predating our study (e.g., in patient BL10). Overall, these findings indicate that many hypnozoites were present in the liver of these infected patients and emphasize that successful malaria eradication will require specific elimination of this important reservoir.

**FIG 5:**
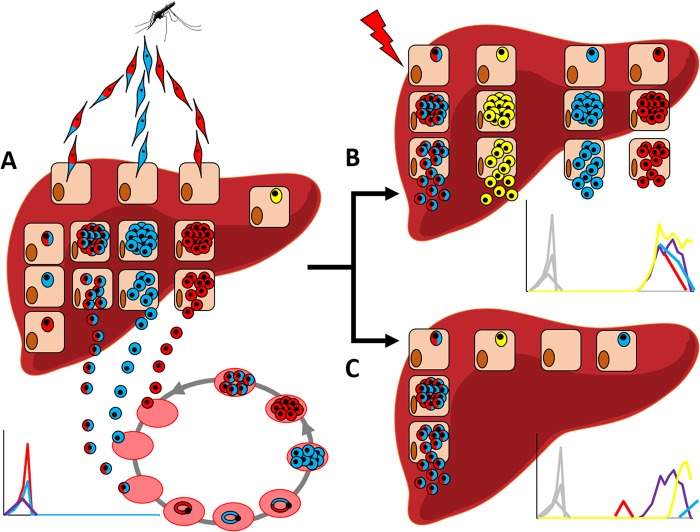
Schematic model of *P. vivax* relapses. (A) Following the bite of an infected mosquito, sporozoites from genetically different *P. vivax* parasites invade hepatocytes, undergo schizogony, and are released in the bloodstream and cause symptoms. A subset of the sporozoites becomes hypnozoites and remain dormant, together with hypnozoites from previous infections (in yellow). (B and C) These hypnozoites then reactivate later, either as an effect of an external stimulus (B) or stochastically (C). Our findings favor the idea of continuous, stochastic reactivation of hypnozoites from the liver (C) as our study did not reveal any stimulus whereas we observed new clones emerging during the course of the relapses.

Beyond enabling rigorous assessment of the prevalence of relapses, our report also provides a unique opportunity to preliminarily assess the biological mechanisms underlying this phenomenon. The observation that many relapses were polyclonal, at the earliest time of genotyping, could suggest that an external stimulus triggers the reactivation of several different hypnozoites at once (Fig. 5). Fever and other infections have been hypothesized to contribute to the activation of hypnozoites (23), but, despite our tight monitoring (which included measurement of axillary temperature every second day), we did not observe any evidence of such a trigger. Alternatively, one could speculate that hypnozoites are constantly reactivating and “leaking” from the liver (Fig. 5). This second hypothesis is supported by the observation that new clones appeared in the bloodstream during the course of several recurring infections (in the absence of reinfections). In addition, our study of CQ efficacy revealed that a sustained presence of parasites was detectable as soon as the blood drug concentration fell below the therapeutic level (while only the sporadic presence of *P. vivax* DNA was able to be detected before) (Popovici et al., submitted for publication). These observations could suggest that reactivation of *P. vivax* hypnozoites occurs continuously but that sustained parasitemia can be observed only once chloroquine has been cleared. While our findings will need to be validated and to be tested in other settings with different transmission levels, the results from this study indicate that the numbers of hypnozoites in infected individuals might be much greater than previously thought.

Finally, while we observed pervasive relapses, the fate of the relapsing clones varied considerably among patients, as did the clinical consequences of the infection; the immune system controlled and eliminated the relapsing parasites in some patients in a few days, one patient remained infected and asymptomatic for several weeks, and five patients developed symptoms of malaria and had to be treated. Such variations illustrate the potential of this study design to provide a rigorous framework not only to study *P. vivax* relapses further but also to understand the pathophysiology of malaria better and to identify host and parasite factors that influence the transition from asymptomatic infection to the development of clinical malaria. Overall, our study data illustrate the promise of modern fieldwork, combined with extensive genomic analyses, for better understanding the biology of *P. vivax* parasites and, hopefully, better controlling vivax malaria.

## MATERIALS AND METHODS

### Patient enrollment and follow-up.

We conducted a prospective clinical drug efficacy study in Ratanakiri Province in Northeastern Cambodia to assess the efficacy of a standard 3-day course of chloroquine (CQ) against vivax malaria. Briefly, febrile patients (or patients with a history of fever in the last 48 h) seeking antimalarial treatment after non-falciparum rapid diagnostic test (RDT)-positive diagnoses (CareStart Malaria HRP2/pLDH Pf/PAN Combo; Access Bio) were offered an opportunity to participate in the study. Exclusion criteria were pregnant or lactating women, signs of severe malaria, known other illnesses, inability to provide informed consent, and age of less than 15 years. We recruited 40 male and female febrile patients from villages within 50 km of the Province capital Banlung and infected solely with *P. vivax* (as determined by real-time PCR). After written informed consent was obtained, all patients received a supervised standard 3-day course of CQ (250 mg). This study was approved by the National Ethic Committee of the Cambodian Ministry of Health (no. 038NECHR) and registered on ClinicalTrials.gov (ClinicalTrials registration no. NCT02118090). Details about the recruitment criteria and patient characteristics are reported elsewhere (Popovici et al., submitted for publication). For the relapse study presented here, we analyzed only patients relocated to a malaria-free area (city of Banlung) for the entire monitoring period (*n =* 20).

Before treatment, we collected 4 ml of blood from each patient. After clearance of the initial infection, which occurred 3 to 5 days after CQ administration (Popovici et al., submitted for publication), we recorded axillary temperature and collected 500 µl of capillary blood from each patient every second day until the end of the monitoring period at day 60. If one patient developed symptoms of malaria during the monitoring period and was confirmed to be positive for *P. vivax* DNA by reverse transcription-PCR (RT-PCR), the patient was retreated by chloroquine (as administered initially) and the monitoring continued for a minimum of 14 days.

### Microscopy and real-time PCR.

We analyzed the parasitemia using Giemsa-stained thick films and estimated the number of parasites per 200 white blood cells (assuming a white blood cell count of 8,000/µl). We extracted parasite DNA from each blood sample by the use of a QIAamp DNA Blood Minikit and determined the presence and species of *Plasmodium* parasites by RT-PCR (24). We also estimated the parasite density for each infection by quantitative PCR (qPCR) using the ratio of *P. vivax* mitochondrial DNA (cytochrome B [24]) to human DNA (β-tubulin [25]) determined by SYBR green qPCR assay. Each PCR amplification was performed in a 20-µl volume with 0.25 µM concentrations of primers, 1× Evagreen SYBR mix, and 1 µl of DNA. All reactions were conducted under the same conditions as follows: 95°C for 15 min followed by 45 cycles of 95°C for 15 s, 60°C for 20 s, and 72°C for 20 s, followed by melting curve analysis.

### High-throughput genotyping of *P. vivax* SNPs.

We genotyped the parasites from all blood samples collected during the initial infections (i.e., before chloroquine treatment) and during all subsequent follow-ups (every second day) using the sequencing-based assay described in reference 16. Briefly, we extracted DNA from each sample and used multiplex PCRs to amplify 100 to 300 bp of DNA sequence surrounding SNPs among Cambodian *P. vivax* strains (15). A total of 128 SNPs distributed throughout the genome were targeted for this study. We pooled all PCR products generated from one sample and used a second PCR to incorporate a unique oligonucleotide sequence. Finally, we pooled the bar-coded PCR products of 96 samples and sequenced them simultaneously on an Illumina MiSeq system to generate 7 to 19 million paired-end reads of 250 bp. We mapped all reads to the Salvador I reference genome sequence (26) using Bowtie2 (27) and used samtool mpileup to summarize the read coverage and allele frequency at each position. For all genotyping analyses, we considered only those nucleotide positions that were covered by at least 100 reads in a given sample (see reference 16 for details).

### Whole-genome sequencing.

We also processed 4 ml of fresh blood collected in EDTA tubes from (i) all patients before CQ treatment and (ii) the patients who, during the follow-up period, developed symptoms of malaria or sustained asymptomatic parasitemia. After leucocyte depletion performed using cellulose columns (28), we isolated DNA from each sample and prepared bar-coded Nextera sequencing libraries according to the manufacturer’s instructions. We then pooled 2 to 6 libraries and sequenced them on one lane of an Illumina 2500 system to generate, on average, 71 million paired-end reads of 100 bp per sample. We mapped all reads to the *P. vivax* reference genome (26) using Bowtie2 (27) and analyzed allelic variations and the relative proportion of each allele for all nucleotide positions covered by at least 50 reads in a given sample. Only samples for which at least 20 million nucleotides were sequenced at more than 50× were considered in the analyses.

### Genetic analyses.

To determine if a given infection was polyclonal, we looked for nucleotide positions with multiple alleles (excluding allele frequencies of <10% as representative of possible sequencing errors [15, 16]) and calculated the most likely number of clones as described in reference 16. We determined the haploid genome sequence of the dominant clone from each infection by using the major allele at each position. Note that this commonly used approach can lead to the presence of artifacts in complex infections with clones in similar proportions (15) and was therefore complemented by additional analyses that are robust with respect to this issue. Finally, we defined “new alleles” as positions that showed intermediate allele frequencies (0.3 to 0.7) in the recurring infections while a single allele was detected in the corresponding initial infection (allele frequency of <0.05 or >0.95).

### Data availability.

The sequence data are freely available in NCBI SRA under BioProject accession no. PRJNA420510.
