# Supplementary material for: Genomic Analyses Reveal the Common Occurrence and Complexity of Plasmodium vivax Relapses in Cambodia
Source: mBio. 2018 Jan 23;9(1):e01888-17. doi: 10.1128/mBio.01888-17 (PMC5784252; doi:10.1128/mBio.01888-17)
Supplement: TABLE S1 [file mbo001183678st1.pdf]

Sample	Time	#Read pairs	#Reads to Pv	%Map Pv	Mean Cov	Cov>50X
BL001	D0	182,251,185	85,052,857	0.23	340	21,470,547
BL002	D0	119,126,773	74,850,388	0.31	299	21,468,797
BL002	D41	59,283,032	30,488,465	0.26	122	21,277,399
BL002	D48	54,680,422	18,611,047	0.17	74	20,748,410
BL002	D55	57,943,411	16,236,946	0.14	65	20,154,194
BL003	D0	79,431,769	78,168,245	0.49	313	21,389,523
BL005	D0	172,545,606	97,429,477	0.28	390	21,518,719
BL006	D0	121,718,373	100,259,252	0.41	401	21,429,996
BL006	D52	55,633,714	45,992	0.00	0	0
BL007	D0	70,697,944	67,794,119	0.48	271	21,446,114
BL007	D43	49,383,712	880,946	0.01	4	1,964
BL007	D51	70,512,473	536,632	0.00	2	688
BL007	D59	67,641,383	1,746,301	0.01	7	7,105
BL008	D0	10,203,766	11,542,471	0.57	46	13,946,644
BL008	D36R	93,727,102	42,719,038	0.23	171	21,042,297
BL009	D0	166,382,152	114,994,939	0.35	460	21,444,419
BL009	D59	56,215,694	10,452	0.00	0	101
BL010	D0	69,448,919	51,309,417	0.37	205	21,326,291
BL010	D56R	111,874,306	44,284,531	0.20	177	21,105,486
BL011	D0	143,883,068	107,744,952	0.37	431	21,503,585
BL011	D48R	50,751,055	36,562,822	0.36	146	20,705,930
BL012	D0	99,069,790	38,324,980	0.19	153	21,279,386
BL012	D47R	146,487,387	94,169,754	0.32	377	21,314,108
BL013	D0	58,540,978	45,894,245	0.39	184	21,305,729
BL014	D0	58,042,285	7,773,758	0.07	31	7,308,680
BL015	D0	73,955,918	11,630,105	0.08	47	16,022,007
BL016	D0	200,138,704	86,966,005	0.22	348	21,469,631
BL016	D49	54,602,235	62,797,962	0.58	251	21,365,981
BL017	D0	90,868,386	66,086,084	0.36	264	21,393,447
BL017	D61	90,643,299	423,421	0.00	2	4,464
BL018	D0	135,631,849	93,470,956	0.34	374	21,299,372
BL018	D58R	59,944,874	40,736,632	0.34	163	21,369,706
BL019	D0	130,442,923	83,577,121	0.32	334	21,436,926
BL020	D0	22,402,713	22,374,784	0.50	89	21,141,965
BL022	D0	47,802,303	23,755,292	0.25	95	20,875,354
BL023	D0	66,843,976	45,107,418	0.34	180	21,090,708
BL023	D53	48,612,297	7,936,360	0.08	32	5,048,631
