# Supplementary figures and images for: Genomic Analyses Reveal the Common Occurrence and Complexity of Plasmodium vivax Relapses in Cambodia
Source: mBio. 2018 Jan 23;9(1):e01888-17. doi: 10.1128/mBio.01888-17 (PMC5784252; doi:10.1128/mBio.01888-17)

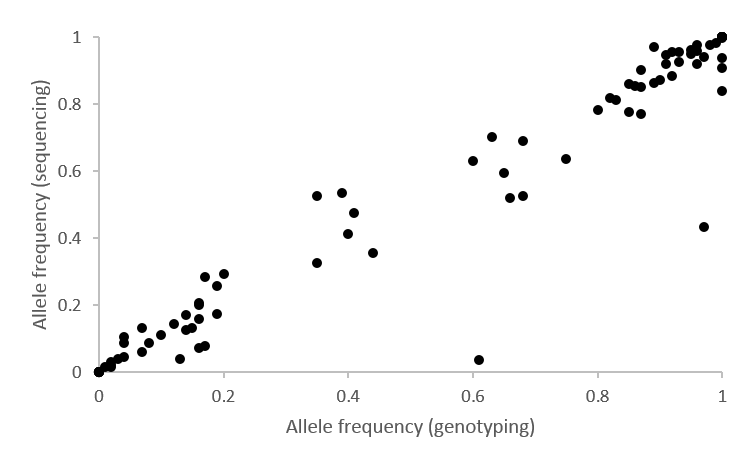

Supplement: FIG S1 [file mbo001183678sf1.tif]

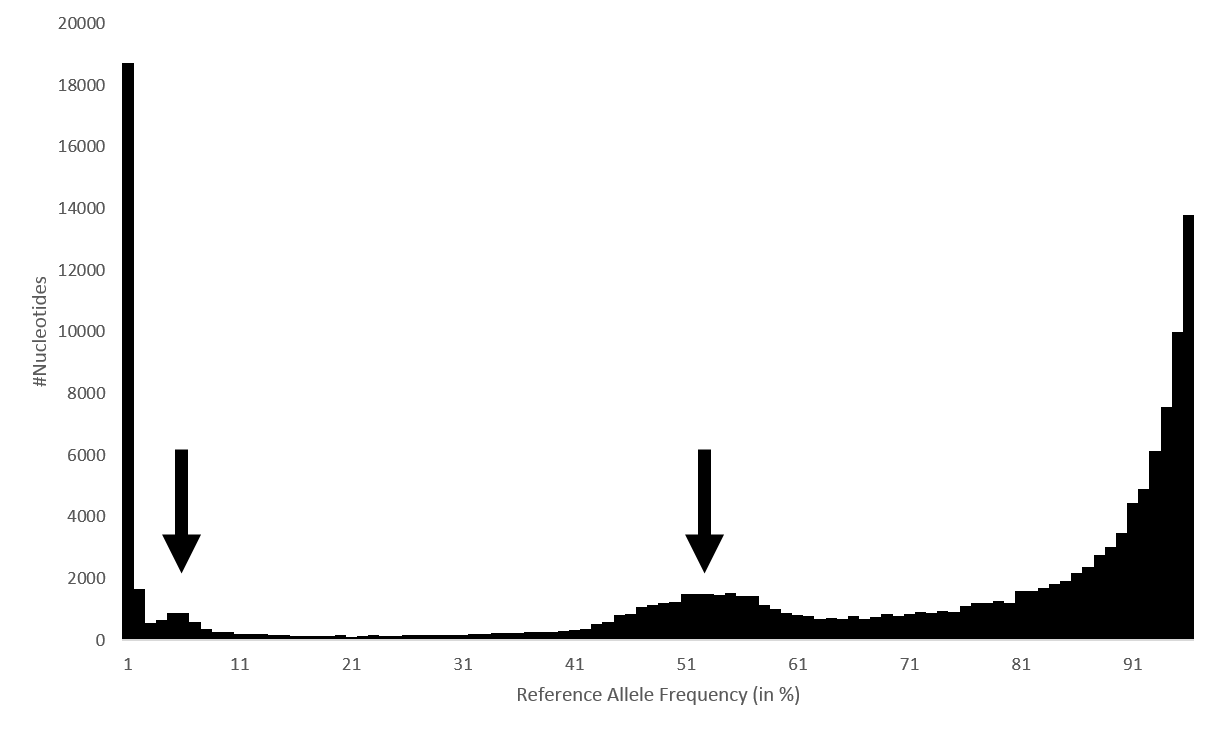

Supplement: FIG S2 [file mbo001183678sf2.tif]

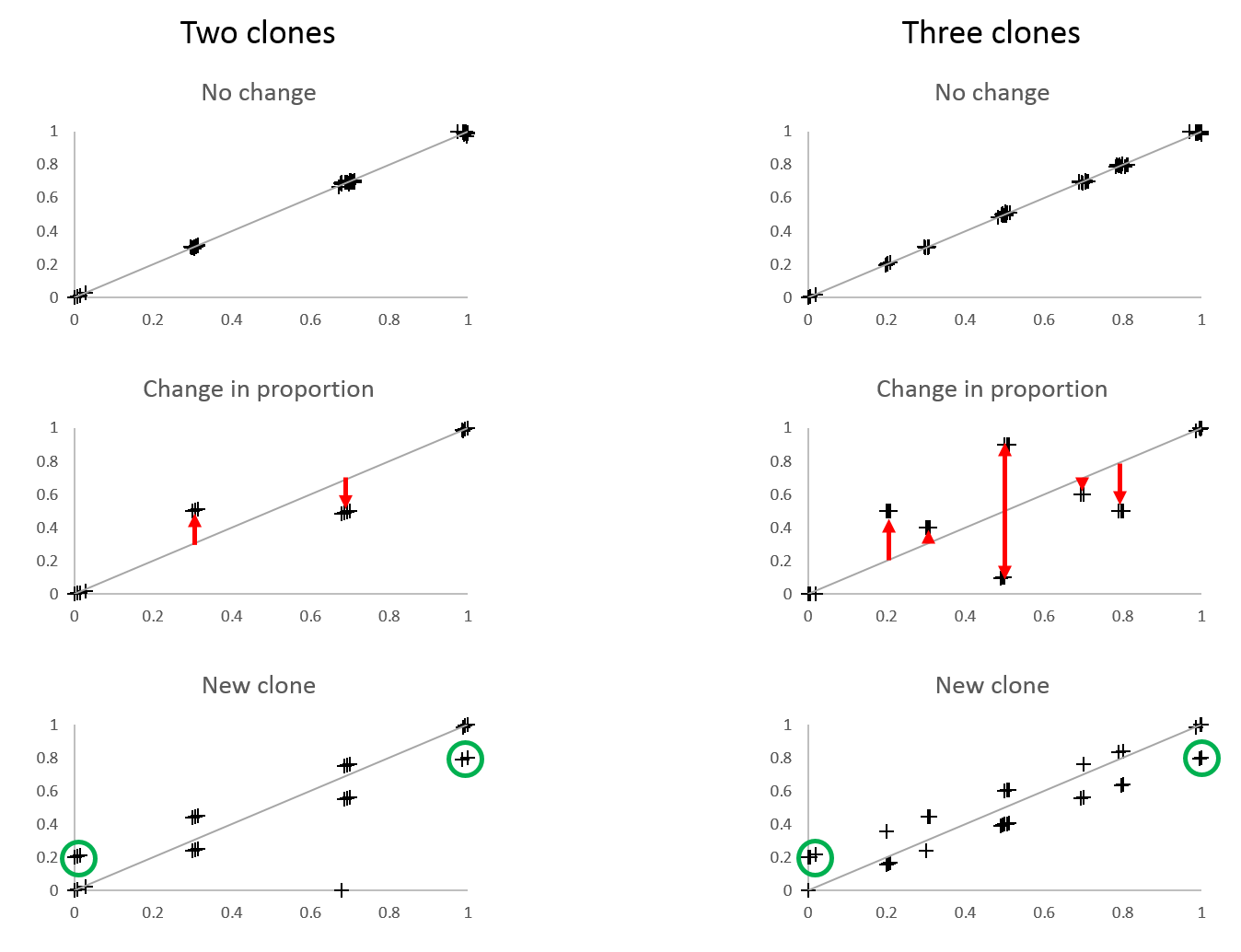

Supplement: FIG S3 [file mbo001183678sf3.tif]

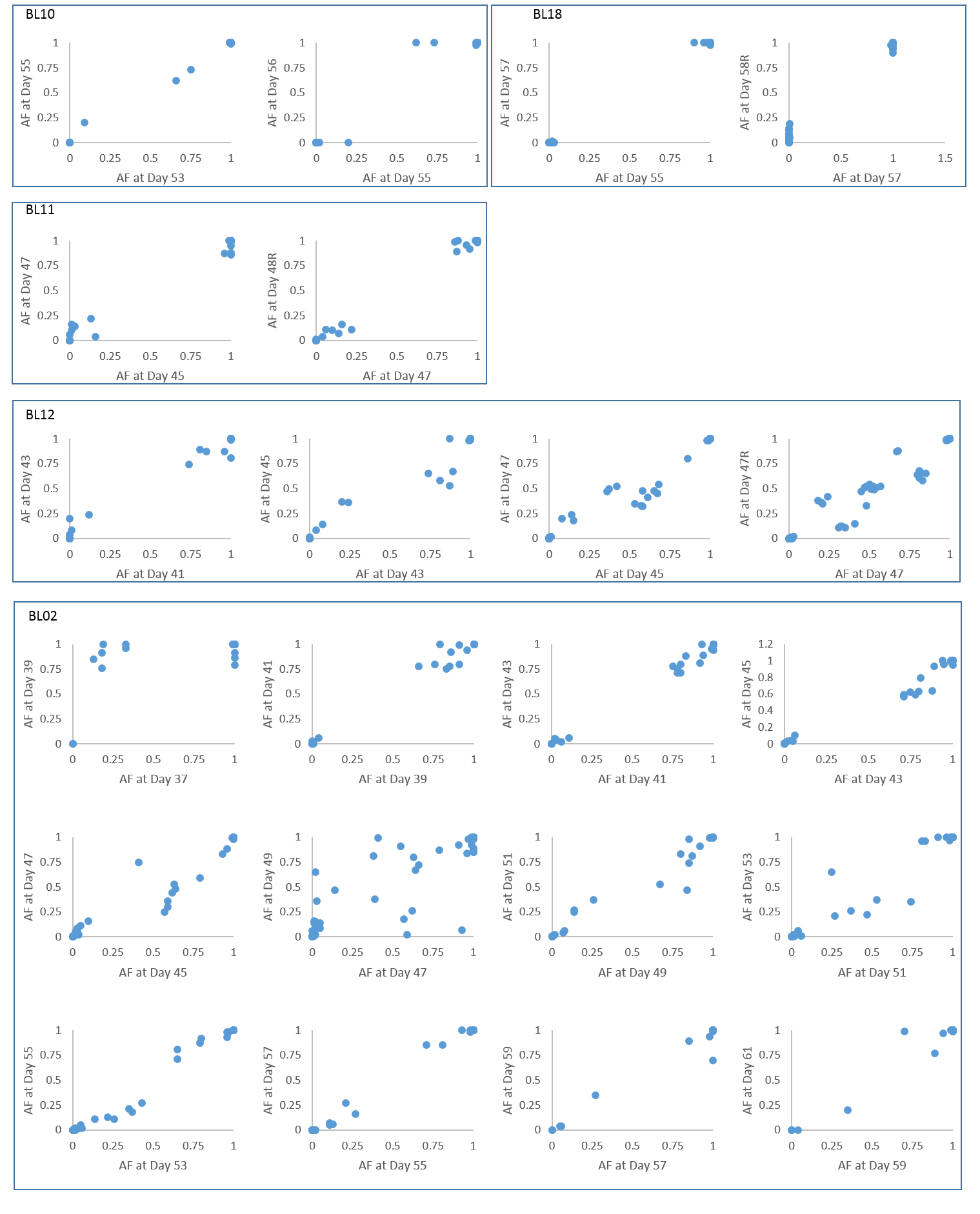

Supplement: FIG S4 [file mbo001183678sf4.tif]
